# Impact of Knife, Disc, and Ball Milling on the Structure and Functionality of Quinoa Flour

**DOI:** 10.3390/foods15020288

**Published:** 2026-01-13

**Authors:** Elias Silva Marcelino, Juan Ignacio González Pacheco, Mariela Beatriz Maldonado, Rocío Miranda Heredia, Alexmilde Fernandes da Silva, Elaine Silva Souza, Thaisa A. S. Gusmão, Heleno Bispo, Rennan P. de Gusmão

**Affiliations:** 1 Department of Food Engineering, Federal University of Campina Grande, R. Aprígio Veloso, 882-Universitário, Campina Grande 58429-900, PB, Brazil; eliassilvamarcelino677@gmail.com (E.S.M.); nandesfera@gmail.com (A.F.d.S.); elaine.souza@estudante.ufcg.edu.br (E.S.S.); thaisa.abrantes@professor.ufcg.edu.br (T.A.S.G.); rennan.pereira@professor.ufcg.edu.br (R.P.d.G.); 2 Department of Chemical Engineering, Federal University of Campina Grande, R. Aprígio Veloso, 882-Universitário, Campina Grande 58429-900, PB, Brazil; heleno.bispo@eq.ufcg.edu.br; 3 Food and Effluent Treatment Laboratory (LATE), Department of Chemical Engineering, Mendoza Regional Faculty, National Technological University, C. Rodriguez 273, Mendoza M5502AJE, Argentina; mbmaldonado@conicet.gov.ar (M.B.M.); rocioheredia098@gmail.com (R.M.H.); 4 CONICET—National Scientific and Technical Research Council, Mendoza Technological Scientific Centre, Av. Ruiz Leal S/N—Parque Gral. San Martín, Mendoza M5502IRA, Argentina

**Keywords:** quinoa flour, milling processes, technological properties, particle size distribution, gelatinisation temperature, food processing, gluten-free formulations, nutritional enhancement

## Abstract

This investigation focuses on optimising the milling processes of white quinoa (*Chenopodium quinoa* Willd.) to enhance its industrial applications. Three milling technologies—knife, disc, and ball milling—were employed to produce flours characterised by various physicochemical analyses. The granulometric analysis indicated that ball milling achieved the finest particle size distribution, significantly improving water absorption capacity and dispersion. Mathematical modelling confirmed that the Rosin–Rammler–Bennett model provided superior predictive capability for rheological behaviour (R^2^ > 0.9624). X-ray diffraction revealed a reduction in crystallinity as milling progressed, while differential scanning calorimetry indicated a decrease in gelatinisation enthalpy and temperature range, suggesting enhanced thermal processing efficiency. Ball milling of the quinoa flour resulted in marked structural changes, as observed by electron microscopy, which are associated in the literature with potential benefits for technological applications in gluten-free and health-oriented foods. Furthermore, fractionation of the flours yielded nutrient-rich bran, containing high levels of protein and fibre. These findings establish critical processing–structure–function relationships, promoting the scalable production of high-value quinoa ingredients that cater to the increasing demand for sustainable and health-oriented food solutions.

## 1. Introduction

Quinoa (*Chenopodium quinoa* Willd.), an ancient Andean pseudocereal from the Chenopodiaceae family, originated in South America and was initially cultivated in Peru and Bolivia. It is consumed by a large part of the Brazilian population. Recent studies highlight the exceptional nutritional profile of quinoa, showing it contains 13–15% protein with all essential amino acids, surpassing traditional cereals [1,2,3,4]. This makes it a valuable option for individuals with coeliac disease due to its natural gluten-free properties. In terms of functionality, quinoa’s various bioactive compounds, such as peptides, polysaccharides, and polyphenols, contribute to its health benefits [5]. Additionally, quinoa’s ability to thrive in extreme environments, exhibiting drought resistance and strong pest resilience, demonstrates its technological advantages. Research on diverse climatic adaptations has further improved these traits through selective breeding [6,7]. Overall, quinoa stands out not only for its dietary benefits but also for its potential in innovative food applications [8,9]. This combination makes it a promising candidate for promoting diet diversification. It demonstrates versatility in diverse culinary and industrial applications, with particular excellence in gluten-free baking, as previously mentioned, due to its high protein content and functional properties. Beyond traditional bread formulations, the flour serves effectively in biscuits, pastries, breakfast cereals, and as a thickening agent in soups and sauces, where its water absorption capacity, ranging from 122 to 295%, depending on particle size, enhances texture and nutritional profiles [10,11,12]. The diverse parameter assessments conducted in this study, including particle size distribution, water absorption capacity, and thermal properties, directly relate to optimising quinoa flour performance across these varied applications, as each application demands specific flour functionalities achievable through appropriate milling processes. Recent investigations have confirmed its superior protein quality, antioxidant potential, and bioactive principles [13], highlighting its role in addressing global food security challenges [14,15,16]. Navruz-Varli et al. [17], studying the nutritional benefits of quinoa for human health, show that due to the quality and quantity of its lipid fraction, quinoa is accepted as an alternative oilseed, presenting an oil content of 2.0% to 9.5%, with this grain being rich in essential fatty acids such as linoleic and alpha-linolenic acids. These fatty acids account for nearly 88% of the total fatty acids in quinoa seeds [18,19,20].

The conversion of quinoa grains into flour is a pivotal processing step that profoundly affects its technological functionality and industrial viability. According to regulatory standards, such as those from Brazil’s National Health Surveillance Agency [21], flours are derived from edible parts of cereals, legumes, fruits, seeds, tubers, or rhizomes through safe technological processes, with the product name specifying the plant species. In the food industry, flours serve as primary, intermediate, or final products, necessitating the preservation of organoleptic and nutritional qualities during production [22]. However, food powders are inherently susceptible to changes in water absorption, softening, fusion, and granulometry due to their composition of living tissues [23,24]. The intensity of milling and grain texture critically determine damaged starch content, influencing technological quality assessments [25]. Milling operations alter grain shape, size, composition through fractionation, and thermal/moisture characteristics [26]. Recent advances underscore how optimised milling preserves bioactive compounds and enhances functional properties in gluten-free formulations [3,13]. For instance, techniques like ball milling reduce crystallinity and improve digestibility while maintaining nutritional integrity [27], and extruded quinoa flour has shown promise in developing gluten-free breads with superior sensory and nutritional profiles [28,29,30,31].

Chenopodium quinoa matrices showed antioxidant, antidiabetic, immunoregulatory, neuroprotective, and antimicrobial effects in in vitro and in vivo models and some clinical studies [32]. Despite quinoa’s increasing commercial relevance amid rising demand for gluten-free and functionally enhanced foods [33], significant knowledge gaps persist regarding the systematic impact of specific milling processes—such as knife, disc, and ball milling—on its flour’s technological properties. While the existing literature emphasises nutritional fortification in cereal products, there is limited exploration of processing–structure–function relationships [18]. Advanced analytical tools are essential for bridging these gaps: mathematical models like Gates–Gaudin–Schuhmann and Rosin–Rammler–Bennett enable precise particle size distribution analysis and process optimisation [34,35,36,37,38,39]. X-ray diffraction elucidates starch crystallinity and polymorphic forms; differential scanning calorimetry characterises thermal transitions like gelatinisation; and scanning electron microscopy reveals morphological features affecting hydration and performance.

This investigation addresses these gaps by systematically evaluating how different milling processes influence the technological properties of white quinoa flour. Through comprehensive analysis using particle size modelling, X-ray diffraction, differential scanning calorimetry, and scanning electron microscopy, the study elucidates key relationships between processing parameters and functional characteristics. The findings offer practical insights for optimising quinoa flour production, enhancing its application in gluten-free and functional foods, and supporting food manufacturers in developing innovative, health-promoting products for diverse markets.

## 2. Materials and Methods

### 2.1. Experimental Location and Facilities

The experiments were conducted at several institutions, including the Laboratory of Food Engineering (LEA) at the Universidade Federal de Campina Grande, located in Campina Grande, Paraíba State, Brazil. Additionally, efforts were made in conjunction with the Unit Operations Laboratory at the Federal University of Paraíba in João Pessoa, Paraíba State, Brazil. Further, this research was undertaken in partnership with the Laboratory of Food and Effluents Treatment (LATE) at the National Technological University, Mendoza Regional Faculty, Mendoza, Argentina.

### 2.2. Raw Materials

#### 2.2.1. Quinoa Grain Procurement and Preparation

**Figure 1:**
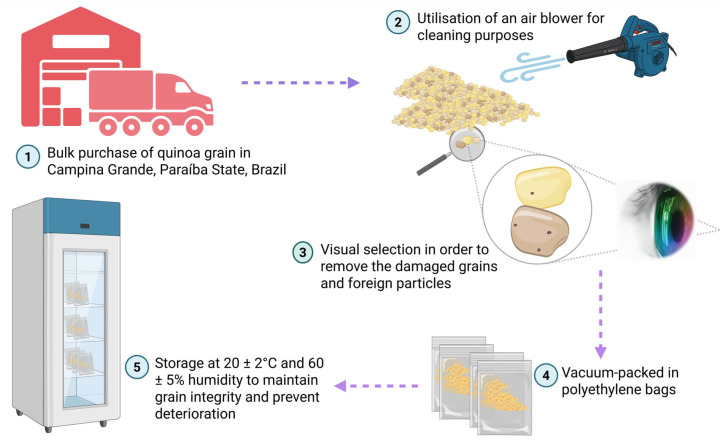
Workflow for the selection, cleaning, and storage of white quinoa grains.

White quinoa grains (*Chenopodium quinoa* Willd.) were procured from local commercial suppliers in the city of Campina Grande, Paraíba State, Brazil. The raw material underwent a selection and preparation protocol consisting of the following sequential steps: initial grain selection through visual inspection to remove damaged, discoloured, or foreign materials; subsequent cleaning using an air blower to eliminate dust, lightweight impurities, and residual debris adhering to the grain surfaces; and storage in vacuum-sealed polyethene bags. The grains were stored in a cool, dry environment at a temperature of 20 ± 2 °C and relative humidity of 60 ± 5% (Figure 1) to preserve grain integrity and prevent deterioration during storage prior to subsequent analyses.

The prepared quinoa grains were maintained under controlled conditions to ensure consistent moisture content and to prevent oxidative changes that could affect the final flour properties. Quality control measures included periodic visual inspection and strict maintenance of optimal storage conditions throughout the experimental period.

#### 2.2.2. Chemicals and Reagents

All analytical grade chemicals and reagents used in this study were obtained from certified suppliers. Sodium hydroxide (NaOH p.a., 1 N standardised solution) was used for acidity titratable determination. Distilled water was utilised throughout all analytical procedures. The gold coating material for scanning electron microscopy sample preparation was of a high-purity grade. The carbon dioxide for critical point drying was of industrial grade with 99.9% purity.

### 2.3. Equipment and Instrumentation

Three distinct milling systems were employed for flour production: (1) knife mill (Marconi, model MA 048, Piracicaba, São Paulo, Brazil), (2) disc mill (Botini, model B5509, Botini Indústria Metalúrgica Ltda., Bilac, São Paulo, Brazil), and (3) ball mill (Tecnal, model TE-350, Tecnal Equipamentos Científicos, Piracicaba, São Paulo, Brazil). Additional equipment included standardised sieves (Tyler series, 42–200 mesh), an electromagnetic sieve shaker (AGT.P-220V Bertel, Bertel Indústria e Comércio de Equipamentos Ltda., Caieiras, São Paulo, Brazil), an analytical balance (precision ± 0.0001 g), a pH meter (calibrated with standard buffer solutions), and a vacuum packaging system for sample storage.

### 2.4. Flour Production and Sample Designation

#### 2.4.1. Milling Process Design and Equipment Specifications

White quinoa grains were processed using three distinct milling technologies to evaluate the comparative effects of different mechanical stress patterns on flour characteristics. The experimental design incorporated three flour samples with specific designations: F1: white quinoa flour obtained through knife milling; F2: white quinoa flour obtained through disc milling; and F3: white quinoa flour obtained through ball milling.

#### 2.4.2. Equipment Technical Specifications

For F1 flour production, a knife mill manufactured by Marconi, model MA 048, was utilised, operating under standardised conditions to ensure reproducible particle size reduction. The F2 flour production employed a disc mill manufactured by Botini, model B5509, configured with optimised disc spacing and rotational parameters for efficient grain fragmentation. F3 flour production utilised a ball mill manufactured by Tecnal, model TE-350, operated with controlled ball-to-grain ratios and a predetermined milling duration to achieve consistent particle size reduction. A summary table is presented below to provide a clear visualisation of the main features and operating conditions of the milling systems utilised in the present study (Table 1).

**Figure 2:**
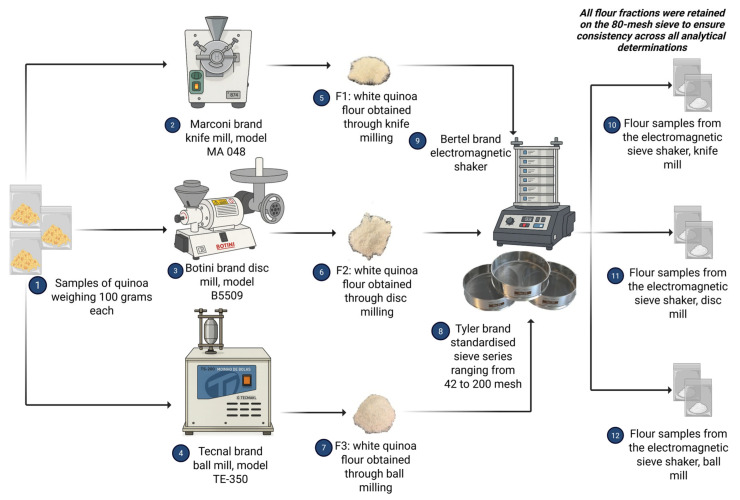
Workflow of the flour production process and sample handling, which illustrates the milling techniques employed in the production of the F1, F2, and F3 flour samples, highlighting the equipment used, including a knife mill (Marconi, model MA 048) for F1 flour, disc mill (Botini, model B5509) for F2 flour, and ball mill (Tecnal, model TE-350) for F3 flour, as well as the post-milling procedure.

Following the milling operations, all flour samples were transferred to vacuum-sealed plastic packages and stored in a dry and cool environment to prevent moisture absorption, oxidative deterioration, and microbial contamination that could compromise subsequent analytical determinations (Figure 2).

### 2.5. Physicochemical Characterisation

#### 2.5.1. Proximate Analysis Methodology

**Figure 3:**
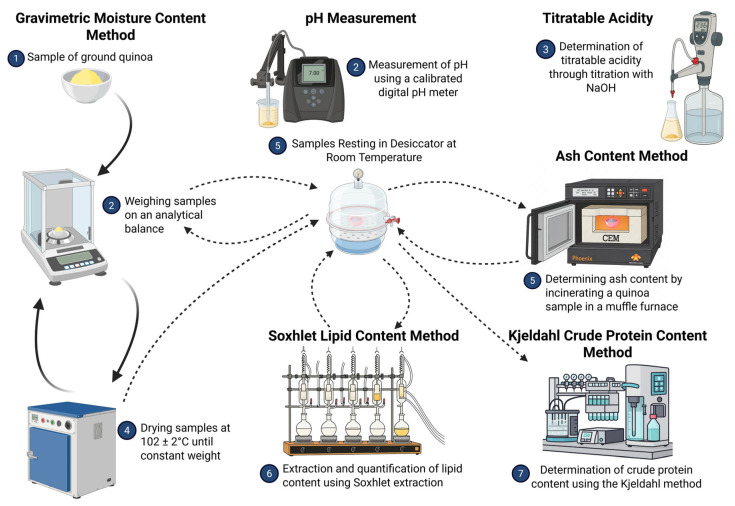
Summary of the tests performed on quinoa grains to analyse key nutritional and compositional factors. The assessment included measuring the moisture, ash, crude protein, lipids, pH, and titratable acidity. The results are expressed in g/100 g of dried matter. Created in BioRender.

Physicochemical analyses were conducted to determine the moisture content, ash content, crude protein, lipids, pH, and titratable acidity following standardised methodologies established by the AOAC standards [40]. These analytical procedures were selected due to their proven reliability and widespread acceptance in food science research applications (Figure 3).

A 100 g sample of quinoa grains, taken representatively from the grain lot, was ground in a mortar for the subsequent determination of its chemical composition. Moisture content determination was performed using the gravimetric method, with oven drying at a controlled temperature of 102 ± 2 °C, until a constant weight was achieved. Ash content was quantified through complete incineration in a muffle furnace under standardised temperature and time conditions. The crude protein content was determined using the Kjeldahl method, with the appropriate nitrogen-to-protein conversion factors applied. Lipid content was extracted and quantified using Soxhlet extraction procedures with appropriate organic solvents. pH measurements were conducted using a calibrated digital pH meter after preparing flour–water suspensions (1:10 *w*/*v*). Titratable acidity was determined by titration with a standardised 1 N NaOH p.a. solution, with results expressed as mL NaOH 1 N/100 g sample [40].

#### 2.5.2. Sieve Analysis Procedure

Particle size distribution analysis was conducted through differential sieving of 100 g white quinoa flour samples, characterised by direct weighing measurements of sieve fractions using a standardised sieve series ranging from 42 to 200 mesh (Figure 2). The sieving operation employed an electromagnetic shaker manufactured by Bertel, operated for a total analysis time of 10 min per sample according to the validated methodology described by Gusmão et al. [41].

The sieve analysis protocol involved weighing each sieve fraction to determine the mass distribution across different particle size ranges. Quality control measures included triplicate analyses and verification of complete mass recovery to ensure analytical accuracy and precision.

#### 2.5.3. Mathematical Modelling of Particle Size Distribution

Two mathematical models were applied, the Gates–Gaudin–Schuhmann (GGS) model and the Rosin–Rammler–Bennett (RRB) model, to evaluate and characterise the particle distribution patterns of the three flour samples F1, F2, and F3. The GGS and RRB models were chosen as they represent established approaches for analysing and modelling particle size distributions in milled food and cereal systems [42,43,44,45].

The Gates–Gaudin–Schuhmann model was expressed according to Equation (1):

(1) $$X_{f}={\left(\frac{a_{n}}{K_{GGS}}\right)}^{I_{GGS}}$$

where $X_{f}$ represents the mass fraction of material finer than the sieve opening $a_{n}$, $K_{GGS}$ is the size parameter representing the average particle size, and $I_{GGS}$ is the distribution parameter representing the dispersion characteristics.

The outcome of the linearisation is as follows, in Equation (2):

(2) $$\ln X_{f}=I_{GGS}\ln\left(\frac{a_{n}}{K_{GGS}}\right)=I_{GGS}\ln a_{n}-I_{GGS}\ln K_{GGS}$$

The Rosin–Rammler–Bennett model, Equation (3), was formulated according to the following:

(3) $$X_{f}=1-e^{-{\left(\frac{a_{n}}{K_{RRB}}\right)}^{I_{RRB}}}$$

where $K_{RRB}$ represents the characteristic size parameter, $I_{RRB}$ represents the uniformity parameter, and $a_{n}$ the sieve opening size, as previously mentioned.

Its linearisation provides the following expression, in Equation (4):

(4) $$\ln\left[-\ln(1-X_{f})\right]=I_{RRB}\ln\left(\frac{a_{n}}{K_{RRB}}\right)=I_{RRB}\ln a_{n}-I_{RRB}\ln K_{RRB}$$

Model parameter determination required linearising both mathematical expressions (Equations (2) and (4), respectively) to enable statistical analysis and goodness-of-fit evaluation through the determination of the correlation coefficient.

#### 2.5.4. Particle Size Standardisation Protocol VG

To standardise the particle size for subsequent analytical procedures, all following analyses were performed exclusively with the flour fraction retained on the 80-mesh sieve from the particle size analysis (Figure 2) [46,47,48,49]. This standardisation approach ensured consistency across all analytical determinations and eliminated potential variations attributable to particle size differences.

### 2.6. Technological Properties’ Characterisation

#### 2.6.1. Scanning Electron Microscopy (SEM) Analysis

**Figure 4:**
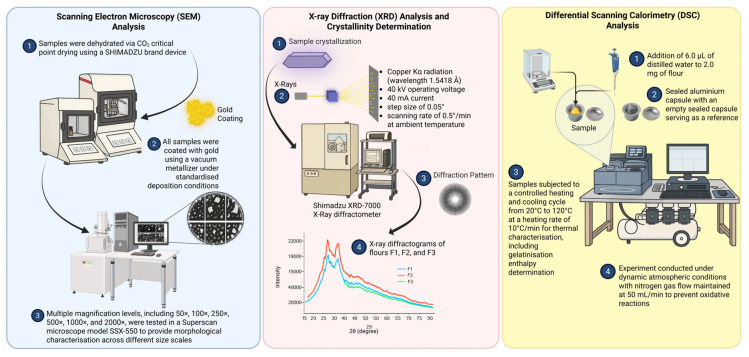
Morphological and structural analysis of flour particles conducted through scanning electron microscopy (SEM), X-ray diffraction (XRD), and differential scanning calorimetry (DSC). Created in BioRender.

Morphological characterisation of flour particles was conducted using scanning electron microscopy following the methodology of Atrous et al. [50], with specific modifications to optimise sample preparation and imaging conditions. The sample preparation protocol involved the following sequential steps: small quantities of flour particles were dispersed on double-sided metallic adhesive tape and mounted on cylindrical metallic supports to ensure representative sampling and optimal particle distribution for microscopic examination (Figure 4).

Additional sample batches were dehydrated using a CO_2_ critical point dryer manufactured by SHIMADZU(Shimadzu Corporation, Kyoto, Japan) to preserve particle morphology and prevent structural artefacts that could compromise morphological analysis. To confer electrical conductivity essential for high-quality imaging, all samples were coated with gold using a vacuum metallizer under standardised deposition conditions.

Micrographic examination was performed using a Superscan model SSX-550 SEM-EDX (Shimadzu Corporation, Kyoto, Japan) operated at an acceleration voltage of 10 kV, with systematic image capture at multiple magnification levels, including 50×, 100×, 250×, 500×, 1000×, and 2000×, to provide morphological characterisation across different size scales.

#### 2.6.2. X-Ray Diffraction (XRD) Analysis and Crystallinity Determination

Crystalline structure analysis was performed using X-ray diffraction employing a Shimadzu XRD-7000 X-ray diffractometer (Shimadzu Corporation, Kyoto, Japan) following the methodology of Won et al. [51], with specific adaptations for quinoa flour analysis. The instrumental parameters were optimised as follows: copper Kα radiation (wavelength 1.5418 Å), 40 kV operating voltage, 40 mA current, step size of 0.05°, and scanning rate of 0.5°/min at ambient temperature (Figure 4).

The diffraction scanning range was adjusted for angles from 5° to 65° (2θ) to capture all relevant crystalline reflections characteristic of starch polymorphs. The obtained diffractograms were utilised for phase identification of crystalline components present in the flour samples. Diffractometer data acquisition was performed through graphical recording, providing counts per second measurements (proportional to diffracted intensity) versus diffraction angle 2θ.

The degree of crystallinity of samples was quantitatively determined according to Equation (5):

(5) $$X_{c}=\frac{I_{c}}{I_{c}+I_{a}}100$$

where $X_{c}$ represents the degree of crystallinity expressed as a percentage, $I_{c}$ is the sum of crystalline peak areas obtained through peak deconvolution, and $I_{a}$ represents the amorphous halo area determined through baseline subtraction methods.

#### 2.6.3. Differential Scanning Calorimetry (DSC) Analysis

Thermal characterisation was performed using differential scanning calorimetry to determine the gelatinisation properties and thermal transitions of the flour samples. Sample preparation involved adding 6.0 μL of distilled water to 2.0 mg of flour in specialised DSC crucibles (aluminium pans) to achieve sample hydration for thermal analysis.

The sealed containers were weighed to verify the accurate mass of the sample and water, then maintained at 30 ± 2 °C for 24 h to ensure complete equilibrium between the flour samples and water. This equilibration period was critical for obtaining reproducible thermal transition temperatures and enthalpies.

Subsequently, the samples were subjected to a controlled heating and cooling cycle from 20 °C to 120 °C at a heating rate of 10 °C/min for comprehensive thermal characterisation, including gelatinisation enthalpy determination. Sealed aluminium capsules were employed, with an empty sealed capsule serving as a reference to eliminate instrumental artefacts and ensure accurate heat flow measurements.

The experiment was conducted under dynamic atmospheric conditions, with nitrogen gas flow maintained at 50 mL/min to prevent oxidative reactions and ensure reproducible thermal behaviour. In addition to enthalpy determinations, the following critical thermal parameters were quantified: onset temperature (T_0_), representing the initial gelatinisation temperature, and peak temperature (T_P_), corresponding to the maximum gelatinisation rate.

#### 2.6.4. Statistical Analysis

Statistical analyses were performed using BioRender Graph^©^ 2024 (Science Suite Inc., Toronto, ON, Canada), adhering to a significance threshold of *p* < 0.05. Prior to conducting any analyses, the normality of the data was evaluated using the Shapiro–Wilk test, while the homogeneity of variance was assessed with Levene’s test. For datasets which demonstrated a normal distribution with equal variances, parametric analyses were applied utilising one-way ANOVA, followed by Tukey’s multiple comparisons test. This approach was specifically employed for the measurements of water content, pH, crude protein content, and lipid content in quinoa grains and flour samples (*n* = 15). When the normality assumptions were not met, non-parametric analyses were conducted using the Kruskal–Wallis test in conjunction with Dunn’s multiple comparisons test, particularly for acidity measurements in flour samples (*n* = 5). The statistical analysis of the data presented in Table 2 (*n* = 3) was conducted utilising IBM^®^ SPSS^®^ Statistics (V22.0, IBM^®^ Corporation, New York, NY, USA).

## 3. Results and Discussion

### 3.1. Grain Characterisation

**Figure 5:**
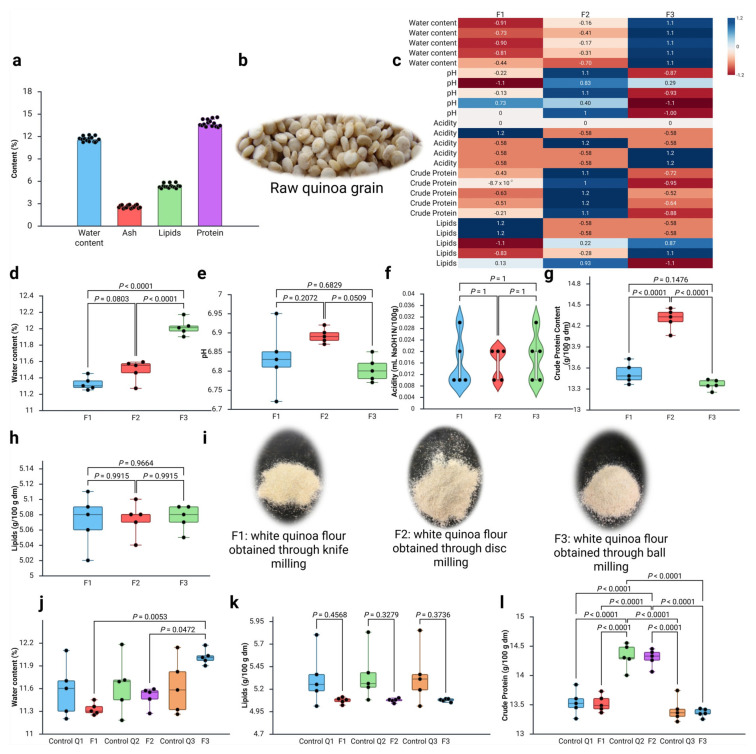
(**a**–**l**) Physicochemical characterisation of raw white quinoa grains and white quinoa flours processed using various milling techniques. (**a**) Proximal composition of quinoa grains showing the water content, ash, lipid, and crude protein percentages (*n* = 15). (**b**) Raw quinoa grains used as starting material. (**c**) Correlation heatmap displaying Pearson correlation coefficients between physicochemical parameters across all flour samples, with the colour intensity representing correlation strength (red: positive correlations, blue: negative correlations). (**d**–**h**) Comparison between the water content (significant differences, F (2, 12) = 58.091, *p* < 0.001), pH (non-significant differences, F (2, 12) = 3.689, *p* = 0.056), titratable acidity (non-significant differences, Kruskal–Wallis statistic = 0.257, *p* = 0.879), crude protein (significant differences, F (2, 12) = 77.814, *p* < 0.001), and lipid content (non-significant differences, F (2, 12) = 0.031, *p* = 0.969) between flour types F1 (knife milling), F2 (disc milling), and F3 (ball milling). (**i**) Visual appearance comparison of white quinoa flours processed using different milling techniques. From left to right: F1 (knife milling), F2 (disc milling), and F3 (ball milling). (**j**–**l**) Comparative analysis between control samples and processed flours showing moisture (significant differences, F (5, 24) = 3.646, *p* = 0.014), lipid (non-significant differences, F (5, 24) = 2.356, *p* = 0.071), and crude protein content (significant differences, F (5, 24) = 33.209, *p* < 0.001), *n* = 5 for parametric and non-parametric analyses, *p* < 0.05 considered statistically significant.

White quinoa grains (*Chenopodium quinoa* Willd.) (Figure 5b) were characterised for their physicochemical composition, revealing a water content of 11.62 ± 0.34%, ash content of 2.56 ± 0.20 (g/100 g dm), protein content of 13.75± 0.46 (g/100 g dm), and lipid content of 5.34 ± 0.28 (g/100 g dm) (Figure 5a). These values align with recent studies highlighting quinoa’s nutritional variability influenced by processing and environmental factors [8,52,53]. The water content falls within the typical range of 10–13% for raw grains, which is crucial for storage stability, with levels below 12% recommended to prevent oxidation and microbial growth. The ash content indicates substantial mineral richness, providing insights into the product’s nutritional density, particularly for bone and metabolic health benefits [15].

In Figure 5c, a correlation heatmap is presented that summarises the linear associations among the physicochemical variables measured across the quinoa flour samples produced by the three milling methods (knife, disc, and ball). Each cell displays a Pearson correlation coefficient (r), with the colour intensity and hue indicating both the magnitude and direction. The heatmap reveals distinct clusters of positively correlated parameters, indicating variables that respond coordinately to milling intensity. Titratable acidity exhibits minimal correlation with the other measured parameters, suggesting that the flour’s buffering capacity remains relatively stable across different milling treatments.

### 3.2. Characterisation of White Quinoa Flour

In Figure 5d–h, the characterisations of flours F1, F2, and F3 for water content, pH, acidity, crude protein content, and lipids are displayed. It can be observed that the average water content for flours F1, F2, and F3 are 11.33 ± 0.08%, 11.49 ± 0.13%, and 12.02 ± 0.10%, respectively (Figure 5d). The pH values are 6.83 ± 0.08, 6.89 ± 0.02, and 6.80 ± 0.03 for F1, F2, and F3, respectively (Figure 5e). Regarding acidity values, these are 0.02 ± 0.01 mL 1 N NaOH/100 g for all quinoa flours (Figure 5f). The protein content of the flours is as follows: flour F1 has a protein content of 13.52 ± 0.14 g/100 g dry matter (dm), flour F2 contains 14.30 ± 0.15 g/100 g dm, and flour F3 exhibits a protein content of 13.36 ± 0.07 g/100 g dm (Figure 5g). Variation in protein content among quinoa flours produced by different milling methods may arise from differences in matrix disruption during processing. More intensive milling can increase the exposure and extractability of protein fractions that are otherwise embedded within the cellular structure, thereby enhancing their quantification in analytical assays. Consequently, the observed differences in measured protein content likely reflect the accessibility of proteins post-milling, rather than compositional changes in the raw material itself. The lipid content for these flours is consistently measured at 5.07 ± 0.03 g/100 g dm for F1, 5.07 ± 0.02 g/100 g dm for F2, and 5.08 ± 0.02 g/100 g dm for F3 (Figure 5h). Additionally, the ash content remains uniform across all flours at 2.78 ± 0.02 g/100 g dry matter (dm).

The crude protein content in the flours ranged from 13.36 to 14.30 g/100 g dry matter (dm), with flour F2 showing a significant difference compared to F1 and F3, indicating a higher crude protein index. These are also similar to those reported by Nowak et al. [53], who found protein levels ranging from 9.1 to 15.7 g/100 g dry matter (dm) in quinoa. Today, quinoa is recognised for its high protein content, which features a balanced amino acid profile with elevated levels of lysine and methionine [54,55,56]. Compared to proteins found in cereals and legumes, the proteins in quinoa have sparked interest in the scientific community regarding the nutritional potential of quinoa [57]. The lipid content found in the flours was, on average, 5.75 g/100 g dry matter (dm). Concerning Figure 5i, the visual appearance of white quinoa flours processed by different milling techniques is represented.

On the other hand, a comparative analysis between control samples and processed flours is presented in Figure 5j–l, which provide insights into the magnitude of compositional changes induced by mechanical milling processes relative to the original quinoa grain matrix. The water content comparison (Figure 5j) reveals statistically significant differences between control and processed samples, with F (5, 24) = 3.646, *p* = 0.014. The significant structural differences between whole quinoa grains and milled flours preclude direct comparisons of moisture content, as observed reductions in flour are more plausibly attributed to processing conditions such as frictional heat generated during milling. The processed flours exhibit a reduced water content compared to control samples, indicating that the mechanical disruption of cellular structures compromises the natural water-holding capacity of the grain matrix [58,59,60]. The lipid analysis (Figure 5k) demonstrates non-significant differences between the control and processed samples, with F (5, 24) = 2.356, *p* = 0.071, indicating that mechanical milling processes preserve the lipid fraction integrity across different processing intensities. The crude protein analysis (Figure 5l) reveals significant differences between control and processed samples, with F (5, 24) = 33.209, *p* < 0.001, representing a significant compositional change induced by mechanical milling. Additionally, the physical breakdown of cellular compartments may redistribute protein fractions, concentrating higher-quality proteins from the embryo and aleurone layers throughout the flour matrix [61,62,63,64]. 

### 3.3. Granulometric Analysis of White Quinoa Flour

**Figure 6:**
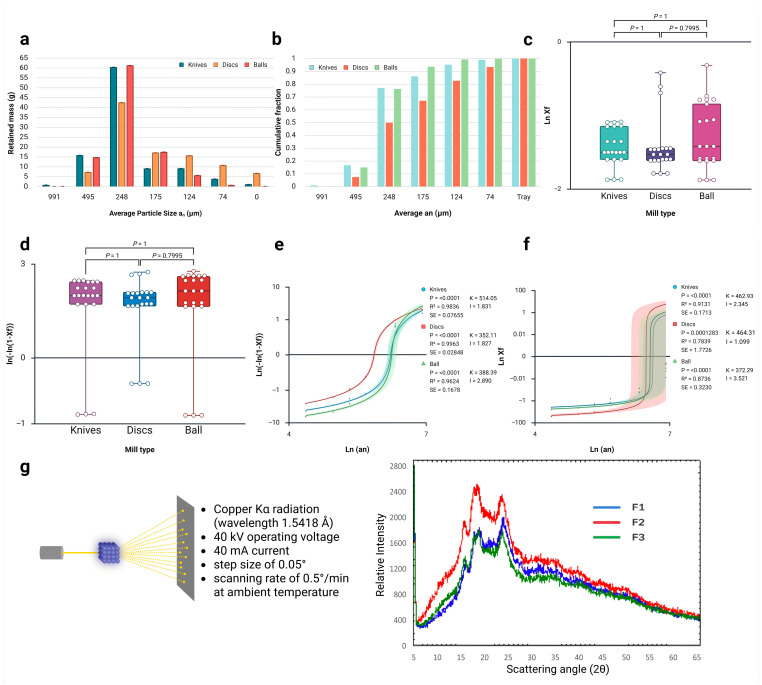
(**a**–**g**) X-ray diffraction and microstructural characterisation of quinoa flours produced by three milling technologies. (**a**) Histogram showing the particle size distribution as a function of the retained mass in grams for quinoa flour produced by different milling methods. (**b**) Histogram of cumulative particle size fractions. (**c**) Comparative distributions of $\ln X_{f}$ and (**d**) $\ln\left(-\ln\left(1-X_{f}\right)\right)$ values across mill types shown with medians, interquartile ranges, and individual data points; brackets denote pairwise comparisons with *p*-values. Non-significant differences for both approaches, GGS and RRB, were detected, with a Kruskal–Wallis statistic = 1.235, *p* = 0.539. Statistical significance was set at *p* < 0.05. (**e**,**f**) RRB (**e**) and GGS (**f**) modelling of cumulative passing: linearised relationships of $\ln\left(-\ln\left(1-X_{f}\right)\right)$ versus $\ln a_{n}$ with fitted curves, 95% confidence intervals, coefficient of determination (R^2^), standard error (SE), and model parameters, such as IGGS and IRRB, as well as KGGS and KRRB, for each mill. In subfigures (**e**,**f**), the colored lines illustrate the fitted curves derived from the experimental data corresponding to each milling method. The blue line corresponds to knife milling, the red line to disc milling, and the green line to ball milling, with each curve serving as the best-fit approximation for its respective dataset. (**g**) X-ray diffraction patterns (5–65° 2θ) of F1, F2 and F3, showing progressive peak broadening and attenuation from knife to ball milling, consistent with a partial loss of crystalline order and increased amorphisation under higher mechanical energy. Created in BioRender.

The experimental data for the sieving of white quinoa flour obtained from knife, disc, and ball mills are presented in Table 2. The retained mass fractions differed among the flours, with the knife-milled flour (F1) exhibiting a higher percentage of larger particles, particularly on the 16-mesh sieve, in contrast to the disc-milled flour (F2), which produced finer particles (Figure 6a).

According to Brazilian legislation, approximately 98% of wheat flour must pass through a sieve with a 250 µm (0.25 mm) mesh opening to be classified as fine flour [21]. The results from granulometric analysis of white quinoa grains fragmented using different mills (knife, disc, and ball) indicate that only the material passing through the 60-mesh sieve (0.248 mm) can be considered fine flour (F). As there is no specific legislation for white quinoa flour, the wheat grain flour regulations were used as a reference, given their commonality across the country [54,65].

Table 2 presents a comparative analysis of the mass retained on each sieve by mill type, along with the transformed variables essential for modelling the accumulated pass-through using the Rosin–Rammler–Bennett (RRB) and Gates–Gaudin–Schuhmann (GGS) processes. All three mills demonstrate a mode around 248 µm (60 mesh), yet they exhibit variations in amplitude and fines generation.

The blade mill (F1) exhibits a concentrated particle size distribution, retaining 60.39 ± 0.231 g on the 60-mesh (248 µm) screen, which comprises 60.3% of the total mass. This concentration in the modal size indicates that the shear mechanism effectively facilitates controlled fragmentation. The formation of ultrafine particles is minimal, with only 1.09 g (1.1%) collected in the tray, confirming that the operation primarily utilises shear forces to promote uniform fragmentation without excessive fineness [66,67].

Conversely, the disc mill (F2) presents heterogeneous behaviour, resulting in a broad particle size distribution across various fractions. The retention on the 60-mesh screen totals 42.53 ± 0.024 g (42.5% of the total mass), along with a substantial range of finer fractions. It is noteworthy that this mill excels in generating ultrafine particles, with a total of 6.72 ± 0.012 g (6.7% of the total mass) collected in the tray. The intermediate fractions account for 43.57 g (43.5% of the total mass), indicating progressive fragmentation influenced by the interplay of impact and shear forces [68].

The ball mill (F3) demonstrates distinct performance characteristics, combining high fragmentation efficiency with effective control over over-pulverisation. It achieves a concentration of 61.29 ± 0.017 g on the 60-mesh screen (61.3% of the total mass), surpassing the blade mill in terms of modal concentration while significantly reducing coarse fractions to 14.83 g (14.8%). A notable attribute of this mill is the minimal production of ultrafine particles, with only 0.12 ± 0.002 g (0.1% of the total mass), attributed to its controlled fragmentation mechanism that efficiently employs both impact and attrition processes [69].

The histogram analysis presented in Figure 6a,b, along with the quantitative modelling data from Table 2, provides insights into the mechanical efficacy and comminution mechanisms inherent to each milling technique. The knife-milled flour (F1) demonstrates retention on coarser sieves (particularly the 16-mesh fraction with 0.780 g retained), indicative of the limited mechanical energy input characteristic of cutting-based size reduction mechanisms. This distribution pattern suggests that the knife milling process operates primarily through shearing forces, with insufficient energy density to achieve particle fragmentation [70,71]. Contrarily, the disc-milled flour (F2) exhibits intermediate particle size reduction efficiency, with reduced retention on the 16-mesh sieve (0.040 g) and enhanced fine particle generation. This intermediate performance reflects the combined action of impact and shearing forces characteristic of disc milling systems, where particle–impactor interactions generate sufficient energy for moderate size reduction whilst maintaining process efficiency. The ball-milled flour (F3) demonstrates the most extensive particle size reduction, with minimal coarse particle retention (0.090 g on 16-mesh) and fine particle generation evidenced by increased tray collection (6.720 g for F2 compared to 0.120 g for F3). This distribution pattern corroborates the high-energy nature of ball milling, where repeated collision and attrition forces generate the mechanical energy necessary for comprehensive particle fragmentation [72,73,74].

Among physicochemical characteristics, granulometry holds significant importance for technological applications, as particle size influences various quality parameters, such as water absorption in dough and the final product’s appearance. The milling process causes tissue disruption, which may allow the internal parts of quinoa to come into contact with air, thus affecting its storage stability [29]. Particle size reduction is an important processing step that can impact the nutritional profile, functional, and rheological properties of quinoa flour [27,75]. These modifications enhance quinoa’s versatility in gluten-free products, where smaller particles improve texture and nutrient bioavailability, as evidenced by studies on starch granule clustering and crystallinity [76,77]. The granulometric distribution varied significantly: F1 retained more large particles (16-mesh sieve), while F3 showed finer particles, with 98% passing through 250 µm, classifying it as fine flour according to wheat legislation. Recent studies confirm that ball milling produces nanometric particles (122–295 nm), improving dispersion and water absorption [75]. The amorphous (non-crystalline) starch content increases with the milling process.

This process of nanoscale reduction, achieved through high-energy mechanical actions such as shear and impact, disrupts the integrity of starch granules, resulting in an increased surface area and modified rheological properties. These findings are consistent with existing research on the role of ball milling in enhancing digestibility and functional characteristics [78,79]. This contrasts with F1 and F2, where larger particles (248–991 µm) result in reduced fineness. In a formerly conducted study, depending on both the ball milling time and speed, the particle size first decreased and then increased, the crystallinity, lamellar structure, and short-range ordered structure gradually decreased, and the contact angle gradually increased [80]. A study performed by Deshpande et al. [33] found that smaller sizes in quinoa flour increase swelling capacity and solubility, positively impacting the texture of baked products. Recent studies have investigated the roller milling process and its effectiveness in fractionating nutrient-dense bran, which is rich in protein and fibre, from perisperm fractions. This approach allows for the development of customised formulations that not only enhance nutritional value but also contribute to a reduction in the glycaemic index of the resulting blends [31,81,82]. Additionally, the application of mathematical models (GGS and RRB) showed a better fit for RRB (R^2^ > 0.96) (Figure 6e), aligning with research that used RRB to predict rheological behaviour in pseudocereal flours [3]. The average diameter (388–514 µm) indicates that F2 and F3 are ideal for wheat blends, reducing the glycaemic index by increasing resistant starch [3,27,30,83]. Overall, these milling-induced changes not only optimise quinoa’s techno-functional attributes but also support its application in sustainable, health-promoting foods, with ball milling emerging as an eco-friendly method for producing high-value flour [31,76,84]. Industrially, such processes enable scalable production of quinoa-based ingredients for gluten-free baking, extruded snacks, and fortified products, enhancing market viability and addressing global demands for nutrient-dense, plant-based foods [33].

### 3.4. Application of Mathematical Models

The mathematical models of Gates–Gaudin–Schuhmann (GGS) and Rosin–Rammler–Bennett (RRB) were applied to the granulometric data from the white quinoa flour samples obtained via knife, disc, and ball mills. The model applications are presented in Figure 6e,f, with their respective linearisation. As observed, the RRB model provided the best fit for each of the respective flours. Using the RRB model, the KRRB values, representing the average particle diameters of the white quinoa flours, were determined. The IRRB represents the slope of the line in the graph, yielding values of 1.831, 1.827, and 2.890 for the flours from the knife, disc, and ball mills, respectively. Additionally, the IRRB uniformity parameters indicate that the ball mill produces the most uniform particle distribution among the three methodologies. The granulometric data indicate that the disc and ball mills achieved superior particle size reduction at laboratory scale, producing 50.29% and 23.94% fine flour (passing through the 60-mesh sieve), respectively. Although the literature reports various studies, numerous factors influence mill efficiency, including the speed, frequency, grinding media material and size, atmosphere, temperature, and grinding time [85,86,87]. From the RRB graph equation, KRRB values of 514.05 µm for F1, 352.11 µm for F2, and an average diameter of 388.39 µm for F3 were obtained (Figure 6e). These values approximate the particle size in the most significant mass fraction from the granulometric distribution, which occurred on the 60-mesh sieve.

This is supported by Deshpande et al. [33], who studied applying RRB to model grinding in quinoa, correlating with flow and compaction properties. Recently, Ahmed et al. [75] have demonstrated that intensive grinding alters crystallinity, affecting starch hydration and pasting. This explains why F3 exhibits a smaller diameter, promoting dense starch networks that enhance viscous textures [27,31]. Milling significantly reduces particle size, which directly enhances functional attributes like water absorption, swelling power, and solubility [27,88]. Smaller particles increase surface area, leading to improved water interaction and bioavailability of nutrients. For instance, ball milling can produce nanoscale particles (122–295 nm), boosting dispersion and hydration. This is particularly evident in finer flours (e.g., F3 from ball milling), where 98% of particles pass through 250 µm, resulting in a higher swelling capacity and solubility compared to coarser fractions from knife or disc milling. All these factors are significant because their relationship underpins the utilisation of quinoa in gluten-free products, where improved hydration enhances texture and dough consistency. It is pertinent to cite the energy dissipation during milling, which disrupts starch granules and elevates solubility, e.g., from 1.5% to 29.7% at higher temperatures [85].

### 3.5. Scanning Electron Microscopy (SEM)

**Figure 7:**
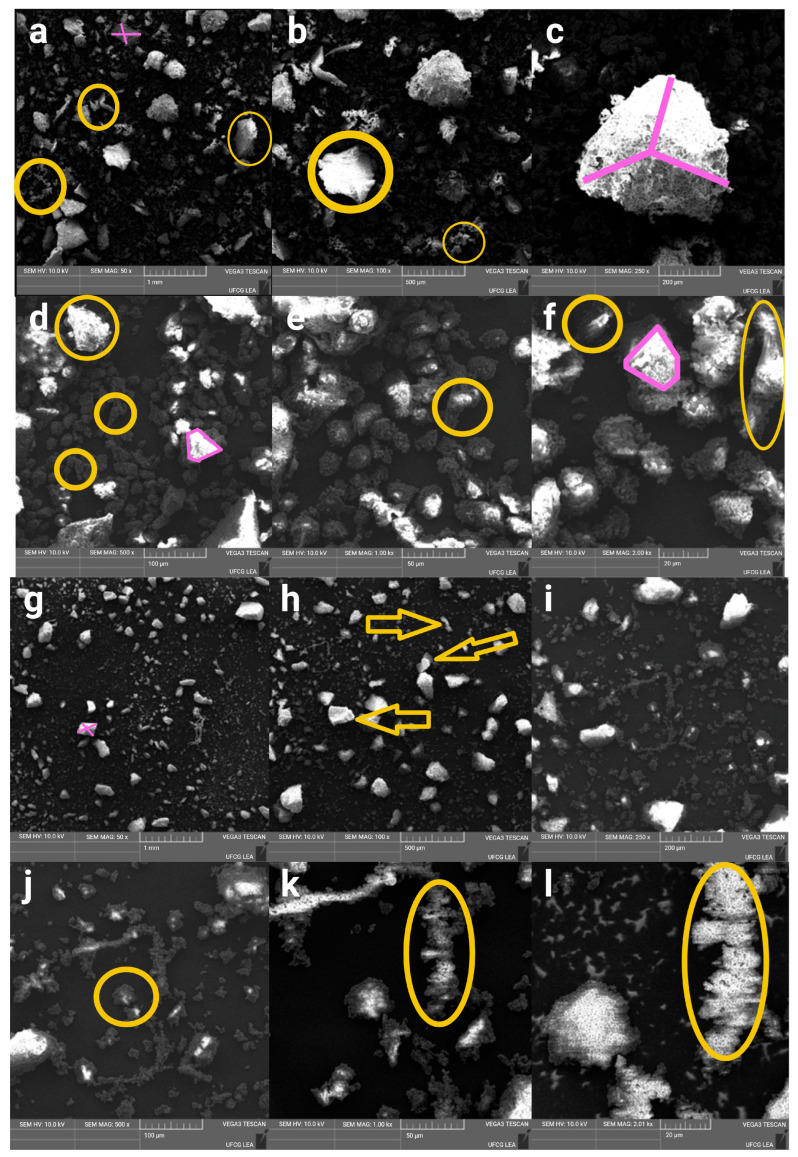

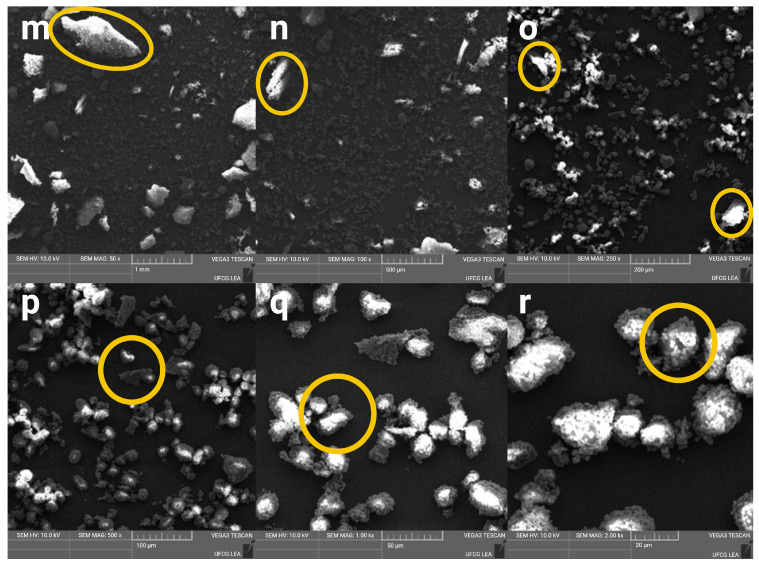
(**a**–**r**) Representative scanning electron micrographs, along with the scale bars, of flours from knife (**a**–**f**), disc (**g**–**l**), and ball (**m**–**r**) mills at increasing magnifications, highlighting differences in particle morphology, fracture surfaces, and aggregation. Magnifications are 50×, 100×, 250×, 500×, 1000×, and 2000×. Yellow arrows and circles indicate regions where granule disruption is observed, while violet lines denote more regular or polygonal granule forms.

The micrographs illustrated in Figure 7a–r depict the gradual structural modifications achieved through knife, disc, and ball milling processes, respectively. The quinoa flour produced via knife milling exhibits characteristic polygonal starch granules that maintain relatively well-preserved structural integrity (Figure 7a–f). The resulting particles demonstrate a heterogeneous size distribution with distinctly defined angular surfaces, which aligns with prior observations of mechanically processed quinoa starches. The microstructural analysis indicates minimal agglomeration phenomena, suggesting that the knife milling process imparts limited mechanical energy compared to alternative methods. The individual starch granules largely remain intact, showcasing the typical quinoa starch morphology, characterised by polygonal shapes ranging from 0.4 to 2.0 μm [89,90]. The surface texture appears moderately rough, indicative of the initial stages of mechanical deformation without extensive fragmentation.

In relation to the disc-milled sample (Figure 7g–l), it demonstrates intermediate levels of particle size reduction and morphological modification. An increased particle fragmentation relative to knife milling is revealed, with evident surface roughening and the emergence of smaller particle fractions [91]. The mechanical action involved in disc milling generates sufficient shear forces to disrupt the original granule structure while preserving recognisable starch particle morphology [92].

Conversely, the ball-milled quinoa flour (Figure 7m–r) exhibits the most significant structural alterations, consistent with the high-energy mechanical processing characteristic of this technique [93]. Ball milling, particularly high-energy ball milling, causes the most significant structural alterations in quinoa flour [27,94]. This method applies intense mechanical actions including shear, friction, collision, and impact, leading to a substantial reduction in particle size, often into the nanoscale range (e.g., 122–295 nm for quinoa) [27,79]. The micrographs reveal extensive particle fragmentation, resulting in numerous smaller particles and notable agglomeration behaviour. The original polygonal starch granules have been predominantly transformed into irregular, deformed structures, leading to a considerable increase in surface area [95].

The three milling processes examined produce distinct levels of particle agglomeration, with the extent of agglomeration increasing alongside the mechanical energy input during milling. Notably, ball-milled samples display the strongest agglomeration, attributable to the increased surface area and reactivity of smaller particles. This enhanced agglomeration facilitates greater water absorption, as the aggregated particles present more hydrophilic surfaces for interaction with water. The increased water uptake, in turn, directly impacts rheological behaviour, reflecting a robust structure–function correlation. This phenomenon arises from the van der Waals forces acting between individual particles, facilitating the formation of larger aggregates from smaller fractured components [92]. The agglomeration tendencies observed in the scanning electron microscopy (SEM) micrographs align with the particle size distribution data presented in Table 2. Despite the extensive fragmentation achieved through the milling processes, the formation of aggregates results in the retention of larger particle fractions. This dynamic interplay between particle breakage and agglomeration exemplifies the “crushing limit” effect commonly noted in high-energy milling operations. The morphological transformations identified through SEM analysis yield valuable insights into the connection between milling-induced structural changes and the functional properties of quinoa flour [79]. The progressive degradation of granular integrity, observed from knife milling to disc milling and ultimately to ball milling, corresponds with an increase in water absorption capacity, alterations in gelatinisation behaviour, and modifications to rheological properties. The substantial increase in surface area, particularly prominent in the ball-milled sample, enhances the accessibility of bioactive compounds and improves digestibility characteristics by disrupting starch clustering.

### 3.6. Nutritional and Bioactive Enhancements

Quinoa is a pseudocereal celebrated for its excellent nutritional quality and potential to improve global food security, especially in marginal environments [56,96]. Nanoscale reduction via ball milling enhances functional properties like digestibility by disrupting starch clusters and increasing bioactive compound accessibility as proteins, fibres, and polyphenols [27,75,97]. Bran fractions from roller milling are nutrient-dense (up to 20% protein and 24% fibre), reducing antinutritional factors and supporting health benefits like the anti-inflammatory and antidiabetic effects reported by other authors [88,97]. With regard to eco-friendly aspects, ball milling is a green method that minimises waste and energy use while preserving minerals (e.g., sulphur and magnesium) and improving the overall nutritional profile [27,75,98]. 

Concerning the sensory and nutritional qualities of baked products, quinoa flour has gained attention in the food industry for its potential to improve both aspects of baked products, such as breads, biscuits, cakes, and gluten-free items [18]. Other studies highlight its high content of proteins, fibres, minerals, and bioactive compounds, which allow the enrichment of traditional products without significantly compromising flavour or texture [3,4,27,75,99,100]. The incorporation of quinoa flour in baked formulations elevates the nutritional profile, resulting in a protein content increase of up to 30% and a fibre increase of approximately 16% compared to equivalent wheat-based products—when up to 30% wheat flour is replaced with quinoa flour [17,88,100,101,102,103,104,105]. This flour releases phenolic compounds during baking, improving antioxidant activity. In breads fortified with 5–15% quinoa, an increase in polyphenols and a reduction in starch digestibility are reported, benefiting people with diabetes [8,13,100]. Andean indigenous crops have excellent potential as sources of health-promoting bioactive compounds, such as flavonoids, and nutritional properties [96]. These advances enable the development of functional products, such as breads with higher vitamin B content (up to 17.8% of daily thiamine) and lower glycaemic impact.

### 3.7. X-Ray Diffraction (XRD) Analysis and Crystallinity

The X-ray diffractograms of flours F1, F2, and F3 are presented in Figure 6g. X-ray diffraction analysis distinguishes three types of crystallinities in granules, classified as A, B, and C based on their form and crystalline structure, with intensity peaks at diffraction angles 2θ, as presented [106].

Analysing X-ray diffraction patterns offers insights into how starch is organised in quinoa flour samples. As shown in Figure 6g, the diffractograms, along with the crystalline structure data in Table 3, highlight structural changes caused by different levels of mechanical processing during the production of the flour. Analysis of the X-ray diffraction patterns reveals that mechanical milling induces measurable changes in quinoa starch crystallinity, reflecting alterations in the ordered molecular arrangement within the flour. The crystallinity degrees for the three flour samples (F1, F2, and F3) varied slightly with mechanical energy input, confirming that intensive milling disrupts the native crystalline structure. Importantly, quinoa flour displays mixed Type A and Type B polymorphic forms, typical of pseudocereals, which confer distinct functional properties such as swelling power, gelation behaviour, and enzyme susceptibility. These crystallographic features are highly relevant for industrial processing, as they influence performance in gluten-free formulations, the ability to form stable gels, and the overall texture of bakery products. Simplifying the polymorph classification enables a clearer understanding of how milling technology impacts starch structure and, consequently, its technological functionality for food applications [107,108].

According to Londoño-Restrepo et al. [109], the broad peaks exhibited by starches are associated with nanocrystalline structures of amylose or amylopectin, due to the elastic and inelastic contributions to the X-ray diffraction pattern in nanoparticles. X-ray diffraction studies have been used to explain starch granule structure and crystallinity. Depending on their biological origin, amylose-to-amylopectin ratio, and amylopectin branch length, starch granules exhibit three diffraction patterns associated with different crystalline polymorphic forms: type A (cereal), type B (tubers), and type C (crystals, or A and B coexisting in the same granule) [88,110]. Recent studies indicate that grinding reduces crystallinity by up to 10–20%, facilitating gelatinisation. Several studies associate this with amylopectin nanocrystalline structures, improving antioxidant properties [27,75,111].

### 3.8. Effects on Cellular Structure, Granule Disruption, and Size Reduction

Quinoa seeds have a complex cellular makeup, with starch granules (polygonal and ~1–2 μm in size) clustered in the perisperm, surrounded by protein-rich embryo and bran layers rich in fibre and bioactive compounds [27,108,110]. Milling breaks down these structures through mechanical forces like shear, impact, and friction [24]. High-energy milling, such as ball milling, transforms quinoa seeds into nanoscale flour (122–295 nm particles), converting polygonal starch granules into flake-like forms and reducing crystallinity without fully altering the A-type polymorph [27,108]. Wet milling yields higher purity fibre fractions (up to 72%) by separating bran and germ, but it increases granule aggregation and surface roughness compared to dry milling [76].

On the other hand, milling reduces relative crystallinity (e.g., from 29% in enzymatic extraction to 26.8% in alkali methods), disrupting amylose and amylopectin arrangements [99]. This leads to a loss of ordered helical structures, with ball milling causing energy dissipation as heat, further degrading granule integrity [98]. Alterations in the cellular structure directly enhance quinoa’s functional properties, making it more versatile for industrial use. Key effects include hydration and swelling, with smaller particles from milling boosting water absorption and swelling power (e.g., up to 16.4 g/g at 85 °C in high-energy milled starch), as disrupted granules allow better water interaction. Ball milling increases solubility from 1.5% to 29.7%, aiding in gluten-free formulations [80,112].

### 3.9. Differential Scanning Calorimetry (DSC)

DSC enables quantitative determinations, as the peak areas are related to the energy involved in the process. The DSC analysis results are presented in Table 4 for parameters including the material heat capacity, enthalpy variation (ΔH), and temperatures of thermal events.

The differential scanning calorimetry analysis revealed significant processing-dependent modifications in thermal behaviour across the three quinoa flour samples (Table 4). The onset temperatures (T_O_) remained relatively consistent at approximately 50 °C across all treatments, indicating that the initial gelatinisation threshold was not fundamentally altered by mechanical processing. However, marked variations were observed in peak gelatinisation temperatures (T_P_), which decreased progressively with milling intensity: F1 (79.35 °C) > F2 (71.68 °C) > F3 (70.47 °C). The gelatinisation temperature range (T_P_–T_O_) narrowed significantly with increasing mechanical processing intensity, from 29.16 °C in knife-milled flour to 20.39 °C in ball-milled flour. This reduction indicates more uniform and rapid gelatinisation behaviour in mechanically disrupted samples, suggesting enhanced thermal efficiency for industrial processing applications.

The most pronounced differences were observed in gelatinisation enthalpy values, with F1 exhibiting the highest energy requirement (1791 J/g) compared to F2 and F3 (1211 and 1221 J/g, respectively). This reduction of approximately 32% in mechanically processed samples indicates that ball and disc milling pre-disrupt crystalline structures, thereby reducing the thermal energy required for complete starch gelatinisation.

These thermal modifications align with findings by Contreras-Jiménez et al. [97], who reported similar onset temperatures for quinoa flour. As noted by Sharma et al. [113], fine grinding reduces peak gelatinisation temperatures, facilitating processing at lower temperatures and suggesting potential for products requiring minimal thermal impact [100,102]. The enhanced thermal responsiveness of mechanically processed quinoa flour enables energy-efficient processing whilst preserving heat-sensitive bioactive compounds, making these flours particularly suitable for gluten-free applications and low-glycaemic formulations. Compared with other studies, the decreased gelatinisation enthalpy in varieties of quinoa species is related to their varied particle size distribution and starch composition, including the amount of bran fraction [114].

## 4. Conclusions

This study evaluated the effects of different mechanical milling methods on the structural and physicochemical properties of white quinoa flour. Experimental results demonstrated that ball milling yields the finest and most homogeneous particle size distribution, accompanied by a reduction in starch crystallinity and alterations in thermal properties. Knife and disc milling produced coarser flour fractions with higher crystallinity degrees, confirming that milling intensity directly influences starch structural organisation.

These structural modifications impact the technological behaviour of quinoa flour. Specifically, the differences in particle size distribution and crystallinity observed among milling methods suggest varied suitability for food industry applications. Finely milled flour with lower crystallinity is suited for instantised or rapidly dispersible products, whereas flours with a higher crystalline content may benefit bakery formulations requiring specific dough stability and texture characteristics. The data-driven findings substantiate the potential for developing customised quinoa flour ingredients tailored to diverse end-use applications.

Limitations of the current work include the study’s focus on a single quinoa variety and laboratory-scale milling equipment. Future research should extend to multiple cultivars, explore pilot- or industrial-scale milling validation, and investigate direct correlations between flour physicochemical properties and final product sensory or nutritional qualities under commercial production conditions. Such studies will enhance the practical applicability and commercial feasibility of quinoa flour formulations.

## Figures and Tables

**Table 1:** Summary of the structural characteristics and operating parameters of the milling systems employed in this study.

Mill Type	Equipment Model	Blade/Disc/Ball Characteristics	Milling Mechanism	Operational Parameters
Knife Mill	Marconi MA 048	Four straight stainless-steel blades, 110 mm length × 2 mm thickness	High-speed cutting and shearing	3000 rpm. 30 s. 40 g batch
Disc Mill	Botini B5509	Pair of hardened steel discs with concentric teeth	Pressure and friction between fixed and rotating discs	1200 rpm. 2 min. 30 g batch. 0.7 mm disc spacing
Ball Mill	Tecnal TE-350	Stainless steel balls (32 mm diameter)	Impact and attrition under planetary motion	617 strokes/min. 6 min per batch. 10:1 ball-to-sample ratio

**Table 2:** Experimental data pertaining to the particle size distribution of white quinoa flours obtained through the knife, disc, and ball milling processes. The retained mass, expressed in grams, as well as $\ln X_{f}$ and $\ln(-\ln\left({1-X}_{f}\right)$, is presented as mean ± SD (standard deviation) (*n* = 3). ‡ Different lowercase letters in the same column indicate significant differences (*p* < 0.05).

Mill Type	Sieve (Mesh)	$\mathrm{Average}\;\mathrm{Size}\;a_{n}$ (µm)	Retained Mass (g)	$\ln a_{n}$	$\ln X_{f}$	$\ln(-\ln\left({1-X}_{f}\right)$
Knife	16	991	0.780 ± 0.017 ^a‡^	6.90	−0.0078 ± 1.71 × 10^−4^ ^a^	1.5801 ± 0.0040 ^a^
	32	495	15.82 ± 0.001 ^b^	6.20	−0.1719 ± 1.19 × 10^−5 a^	0.6128 ± 3.42 × 10^−5 a^
	60	248	60.39 ± 0.231 ^c^	5.51	−0.9235 ± 0.006 ^a^	−0.6811 ± 0.0080 ^a^
	80	175	9.100 ± 0.079 ^d^	5.16	−0.0952 ± 0.001 ^a^	0.8749 ± 0.0040 ^a^
	115	124	9.160 ± 0.058 ^d^	4.82	−0.0959 ± 0.001 ^a^	0.8721 ± 0.0030 ^a^
	200	74	3.830 ± 0.011 ^e^	4.30	−0.0390 ± 1.14 × 10^−4^ ^a^	1.1830 ± 0.0010 ^a^
	Tray	-	1.090 ± 0.027 ^ae^	-	-	-
Disc	16	991	0.040 ± 0.028 ^a^	6.900	−0.0004 ± 2.80 × 10^−4^ ^a^	2.0574 ± 0.1090 ^a^
	32	495	7.300 ± 0.011 ^b^	6.200	−0.0757 ± 1.18 × 10^−4^ ^a^	0.9628 ± 0.0010 ^a^
	60	248	42.53 ± 0.024 ^b^	5.510	−0.5527 ± 4.16 × 10^−4^ ^a^	−0.1548 ± 0.0010 ^a^
	80	175	17.14 ± 0.032 ^c^	5.160	−0.1877 ± 3.85 × 10^−4^ ^a^	0.5684 ± 0.0010 ^a^
	115	124	15.67 ± 0.090 ^c^	4.822	−0.1701 ± 0.0010 ^a^	0.6179 ± 0.0030 ^a^
	200	74	10.76 ± 0.010 ^bc^	4.300	−0.1136 ± 1.12 × 10^−4^ ^a^	0.8024 ± 4.17 × 10^−4^ ^a^
	Tray	-	6.720 ± 0.012 ^b^	-	-	-
Ball	16	991	0.090 ± 0.084 ^a^	6.900	−0.0009 ± 0.0010 ^a^	1.9479 ± 0.2230 ^a^
	32	495	14.74 ± 0.002 ^b^	6.200	−0.1594 ± 2.34 × 10^−5 a^	0.6498 ± 7.08 × 10^−5 a^
	60	248	61.29 ± 0.017 ^c^	5.510	−0.9481 ± 4.38 ×10^−4^ ^a^	−0.7130 ± 0.0010 ^a^
	80	175	17.47 ± 0.046 ^b^	5.160	−0.1919 ± 0.0010 ^a^	0.5569 ± 0.0020 ^a^
	115	124	5.660 ± 0.008 ^b^	4.820	−0.0582 ± 8.47 × 10^−5 a^	1.0551 ± 4.92 ×10^−4^ ^a^
	200	74	0.690 ± 0.051 ^a^	4.300	−0.0069 ± 0.0010 ^a^	1.6048 ± 0.0150 ^a^
	Tray	-	0.120 ± 0.002 ^a^	-	-	-

**Table 3:** X-ray diffraction analysis and crystalline structure characterisation of quinoa flour samples processed by different milling techniques. Peak positions (2θ°), relative peak intensities (counts), d-spacing values (Å), and assigned crystalline phases show characteristic A-type starch diffraction patterns with major peaks at 15.3°, 17.1°, 18.0°, and 23.1°, and a V-type amylose–lipid complex peak at ~20°. F1: knife milling; F2: disc milling; F3: ball milling.

Types of Crystallinity	Crystallinity Degrees (%)	Intensity Peaks at the Diffraction Angle 2θ
A	18.8	15.3°|17.1°|18.2°|23.5°
B	16.2	5.6°|14.4°|17.2°|22.2°|24°
C	17.7	5.6°|15.3°|17.3°|23.5°

**Table 4:** Differential scanning calorimetry parameters of white quinoa flours obtained through knife (F1), disc (F2), and ball (F3) milling processes. Reported parameters include the following: onset temperature (T_O_, °C); peak temperature (T_P_, °C); gelatinisation temperature range (T_P_–T_O_, °C); and enthalpy of gelatinisation (ΔH, J/g dry basis). All values represent the mean ± standard deviation (*n* = 5 independent measurements from separate batches). ^⁑^ Different lowercase letters within the same row indicate statistically significant differences between treatments (*p* < 0.05).

	Flours		
Parameters	F1	F2	F3
T_O_ (°C) (gelatinisation)	50.19 ± 0.01 ^a⁑^	50.04 ± 0.02 ^b^	50.08 ± 0.02 ^ab^
T_P_ (°C)	79.35 ± 0.03 ^a^	71.68 ± 0.09 ^ab^	70.47 ± 0.02 ^b^
T_P_–T_O_ (°C)	29.16 ± 0.03 ^a^	21.64 ± 0.09 ^ab^	20.39 ± 0.04 ^b^
ΔH (J/g) (enthalpy of gelatinisation)	1791 ± 0.71 ^a^	1211 ± 0.71 ^b^	1221 ± 1.10 ^c^

## Data Availability

The original contributions presented in the study are included in the article, further inquiries can be directed to the corresponding author.

## References

[1] Schmidt D., Mendes M.T., Borges R., De Souza Gallo A., Forti V.A., Verruma-Bernardi M.R. (2024). Purchase and Consumption Habits for Quinoa and Amaranth in Brazil. Food Sci. Technol..

[2] Manzanilla-Valdez M.L., Boesch C., Orfila C., Montaño S., Hernández-Álvarez A.J. (2024). Unveiling the Nutritional Spectrum: A Comprehensive Analysis of Protein Quality and Antinutritional Factors in Three Varieties of Quinoa (*Chenopodium quinoa* Wild). Food Chem. X.

[3] Mu J., Qi Y., Gong K., Chen Z., Brennan M.A., Ma Q., Wang J., Brennan C.S. (2023). Effects of Quinoa Flour (*Chenopodium Quinoa* Willd) Substitution on Wheat Flour Characteristics. Curr. Res. Food Sci..

[4] Ghumman A., Mudgal S., Singh N., Ranjan B., Kaur A., Rana J.C. (2021). Physicochemical, Functional and Structural Characteristics of Grains, Flour and Protein Isolates of Indian Quinoa Lines. Food Res. Int..

[5] Ren G., Teng C., Fan X., Guo S., Zhao G., Zhang L., Liang Z., Qin P. (2023). Nutrient Composition, Functional Activity and Industrial Applications of Quinoa (*Chenopodium Quinoa* Willd.). Food Chem..

[6] Dehghanian Z., Ahmadabadi M., Asgari Lajayer B., Gougerdchi V., Hamedpour-Darabi M., Bagheri N., Sharma R., Vetukuri R.R., Astatkie T., Dell B. (2024). Quinoa: A Promising Crop for Resolving the Bottleneck of Cultivation in Soils Affected by Multiple Environmental Abiotic Stresses. Plants.

[7] Chauhan G.S., Zillman R.R., Eskin N.A.M. (1992). Dough Mixing and Breadmaking Properties of Quinoa-Wheat Flour Blends. Int. J. Food Sci. Technol..

[8] Pathan S., Siddiqui R.A. (2022). Nutritional Composition and Bioactive Components in Quinoa (*Chenopodium Quinoa* Willd.) Greens: A Review. Nutrients.

[9] Stikic R., Glamoclija D., Demin M., Vucelic-Radovic B., Jovanovic Z., Milojkovic-Opsenica D., Jacobsen S.E., Milovanovic M. (2012). Agronomical and Nutritional Evaluation of Quinoa Seeds (*Chenopodium Quinoa* Willd.) as an Ingredient in Bread Formulations. J. Cereal Sci..

[10] Pooja M., Neha M., Ranu P. (2021). Formulation and Characterization of Gluten Free Bread Based on Quinoa and Rice Flour. Int. J. Curr. Microbiol. Appl. Sci..

[11] Vejdanivahid S., Salehi F. (2025). Enhancing the Quality and Nutritional Properties of Gluten-Free Pancakes Using Sprouted Quinoa Flour Treated With Magnetic Fields, Ultrasound, and Infrared Drying. Food Sci. Nutr..

[12] Abdelshafy A.M., Rashwan A.K., Osman A.I. (2024). Potential Food Applications and Biological Activities of Fermented Quinoa: A Review. Trends Food Sci. Technol..

[13] Tanwar B., Goyal A., Irshaan S., Kumar V., Sihag M.K., Patel A., Kaur I. (2019). Quinoa. Whole Grains and their Bioactives: Composition and Health.

[14] Afzal I., Haq M.Z.U., Ahmed S., Hirich A., Bazile D. (2023). Challenges and Perspectives for Integrating Quinoa into the Agri-Food System. Plants.

[15] Gautheron O., Nyhan L., Ressa A., Torreiro M.G., Tlais A.Z.A., Cappello C., Gobbetti M., Hammer A.K., Zannini E., Arendt E.K. (2024). Solid-State Fermentation of Quinoa Flour: An In-Depth Analysis of Ingredient Characteristics. Fermentation.

[16] Guo Z., Deng X., Ping C., Li X., Li D., Wu X., Xiao X., Kong R. (2025). Quinoa: Nutritional and Phytochemical Value, Beneficial Effects, and Future Applications. Appl. Food Res..

[17] Navruz-Varli S., Sanlier N. (2016). Nutritional and Health Benefits of Quinoa (*Chenopodium quinoa* Willd.). J. Cereal Sci..

[18] Abugoch James L.E. (2009). Quinoa (*Chenopodium quinoa* Willd.): Composition, Chemistry, Nutritional, and Functional Properties. Adv. Food Nutr. Res..

[19] Filho A.M.M., Pirozi M.R., Borges J.T.D.S., Pinheiro Sant’Ana H.M., Chaves J.B.P., Coimbra J.S.D.R. (2017). Quinoa: Nutritional, Functional, and Antinutritional Aspects. Crit. Rev. Food Sci. Nutr..

[20] Wang S., Wang M., Zhou Y., Yang R., Chen H., Wu J., Xu J., Tu K., Shi J., Sun X. (2024). Basic Nutrients and UPLC- ZenoTOF-MS/MS Based Lipomics Analysis of *Chenopodium quinoa* Willd. Varieties. Food Prod. Process. Nutr..

[21] ANVISA (2008). Resolução No 91.

[22] Repo-Carrasco-Valencia R., Tomos M.C. (2022). Native Crops in Latin America Biochemical, Processing, and Nutraceutical Aspects.

[23] Rao M.A., Rizvi S.S., Datta A.K. (2014). Engineering Properties of Foods.

[24] Barbosa-Cánovas G.V., Ortega-Rivas E., Juliano P., Yan H. (2005). Food Powders—Physical Properties, Processing, and Functionality.

[25] Scholz M.B.S., Bordignon J.R., de Miranda M.Z., da Silva V.C., Tatsch P.O. Granulometria e Amido Danificado de Farinhas de Trigo Obtidas Em Diferentes Moinhos Experimentais. https://www.alice.cnptia.embrapa.br/alice/handle/doc/1020729.

[26] Coțovanu I., Batariuc A., Mironeasa S. (2020). Characterization of Quinoa Seeds Milling Fractions and Their Effect on the Rheological Properties of Wheat Flour Dough. Appl. Sci..

[27] Ahmed J., Thomas L., Arfat Y.A. (2019). Functional, Rheological, Microstructural and Antioxidant Properties of Quinoa Flour in Dispersions as Influenced by Particle Size. Food Res. Int..

[28] Burbano J.J., Di Pierro J.P., Camacho C., Vidaurre-Ruiz J., Repo-Carrasco-Valencia R., Iglesias F.A., Sánchez M., Ospina Y.A.M., Igartúa D.E., Correa M.J. (2025). Extruded Quinoa Flour Applied for the Development of Gluten-Free Breads: A Technological, Sensory and Microstructural Approach. Plant Foods Hum. Nutr..

[29] Ni D., Gao F., Cao H., Song H., Huang K., Zhang Y., Wang X., Tan Z., Lu J., Guan X. (2025). Moderate Milling Improved Storage Stability of Quinoa Based on the Evaluation of Lipid Oxidation and Physicochemical Characteristics. J. Food Sci..

[30] Saroja P.E., Muthugounder P., Shanmugam S., Dhairiyasamy R. (2024). Enhancing Flour Quality and Milling Efficiency: Experimental Study on Bullet Plate Type Flour Grinding Machine. Matéria.

[31] Wang C., Cao H., Wang P., Dai Z., Guan X., Huang K., Zhang Y., Song H. (2023). Changes of Components and Organizational Structure Induced by Different Milling Degrees on the Physicochemical Properties and Cooking Characteristics of Quinoa. Food Struct..

[32] Casalvara R.F.A., Ferreira B.M.R., Gonçalves J.E., Yamaguchi N.U., Bracht A., Bracht L., Comar J.F., de Sá-Nakanishi A.B., de Souza C.G.M., Castoldi R. (2024). Biotechnological, Nutritional, and Therapeutic Applications of Quinoa (*Chenopodium quinoa* Willd.) and Its By-Products: A Review of the Past Five-Year Findings. Nutrients.

[33] Deshpande R.H., Kumar A., Katare M., Sakhare S.D., Inamdar A.A. (2022). Effect of Grinding Techniques and Supplementation of Quinoa on the Carbohydrate Profile of Tortillas. J. Food Sci. Technol..

[34] Almeida R.L.J., Santos N.C., dos Santos Pereira T., de Alcântara Silva V.M., Silva L.N., Santiago Â.M., Moreira F.I.N., da Silva L.R.I., Borges E.M.E.S., de Queiroga A.P.R. (2020). Análise Morfológica Em Flocos de Arroz. Res. Soc. Dev..

[35] Camalan M. (2021). Investigating the Effects of Random Sieving Losses on Particle Size Distributions. Part. Sci. Technol..

[36] Camalan M. (2020). Simulating Probabilistic Sampling on Particle Populations to Assess the Threshold Sample Sizes for Particle Size Distributions. Part. Sci. Technol..

[37] Camalan M. (2020). Estimating the Number-Weighted Equivalents of the Mass-Weighted Size Distribution Functions. Powder Technol..

[38] Colorado-Arango L., Menéndez-Aguado J.M., Osorio-Correa A. (2021). Particle Size Distribution Models for Metallurgical Coke Grinding Products. Metals.

[39] Miao Y., Zhang Y., Wu D., Li K., Yan X., Lin J. (2021). Rock Fragmentation Size Distribution Prediction and Blasting Parameter Optimization Based on the Muck-Pile Model. Min. Met. Explor..

[40] AOAC (2011). Official Methods of Analysis of the Association of Official Analytical Chemists International.

[41] de Gusmão R.P., Cavalcanti-Mata M.E.R.M., Duarte M.E.M., Gusmão T.A.S. (2016). Particle Size, Morphological, Rheological, Physicochemical Characterization and Designation of Minerals in Mesquite Flour (*Proposis julifrora*). J. Cereal Sci..

[42] Elmisaoui S., Latifi A.M., Khamar L. (2024). Analysis of the Dissolution of Phosphate Ore Particles in Phosphoric Acid: Influence of Particle Size Distribution. Hydrometallurgy.

[43] Stoyan D., Zhang Z.X. (2023). A Stochastic Model Leading to Various Particle Mass Distributions Including the RRSB Distribution. Granul. Matter..

[44] He T., Xu Z., Li Z., Zhao X., Zhao S., Liu Y. (2022). Study on the Relationship between the Particle Size Distribution Characteristics of Ground Granulated Blast Furnace Slag and Its Mortar Properties. Front Mater..

[45] Petrakis E., Komnitsas K. (2022). Modeling of Bauxite Ore Wet Milling for the Improvement of Process and Energy Efficiency. Circ. Econ. Sustain..

[46] Pang J., Guan E., Yang Y., Li M., Bian K. (2021). Effects of Wheat Flour Particle Size on Flour Physicochemical Properties and Steamed Bread Quality. Food Sci. Nutr..

[47] Hu H., Zhou X., Zhang Y., Zhou W., Zhang L. (2023). Influences of Particle Size and Addition Level on the Rheological Properties and Water Mobility of Purple Sweet Potato Dough. Foods.

[48] Katyal M., Virdi A.S., Singh N., Chopra N., Kaur A., Ahlawat A.K., Singh A.M. (2018). Fractionation and Grain Hardness Effect on Protein Profiling, Pasting and Rheological Properties of Flours from Medium-Hard and Extraordinarily Soft Wheat Varieties. J. Food Sci. Technol..

[49] Adzqia F., Suwonsichon S., Thongngam M. (2023). Effects of White Sorghum Flour Levels on Physicochemical and Sensory Characteristics of Gluten-Free Bread. Foods.

[50] Atrous H., Benbettaieb N., Chouaibi M., Attia H., Ghorbel D. (2017). Changes in Wheat and Potato Starches Induced by Gamma Irradiation: A Comparative Macro and Microscopic Study. Int. J. Food Prop..

[51] Won C., Jin Y.-I., Kim M., Lee Y., Chang Y.H. (2017). Structural and Rheological Properties of Potato Starch Affected by Degree of Substitution by Octenyl Succinic Anhydride. Int. J. Food Prop..

[52] De Bock P., Cnops G., Muylle H., Quataert P., Eeckhout M., Van Bockstaele F. (2022). Physicochemical Characterization of Thirteen Quinoa (*Chenopodium quinoa* Willd.) Varieties Grown in North-West Europe—Part II. Plants.

[53] Nowak V., Du J., Charrondière U.R. (2016). Assessment of the Nutritional Composition of Quinoa (*Chenopodium quinoa* Willd.). Food Chem..

[54] Angeli V., Silva P.M., Massuela D.C., Khan M.W., Hamar A., Khajehei F., Graeff-Hönninger S., Piatti C. (2020). Quinoa (*Chenopodium quinoa* Willd.): An Overview of the Potentials of the “Golden Grain” and Socio-Economic and Environmental Aspects of Its Cultivation and Marketization. Foods.

[55] Cervilla N.S., Mufari J.R., Calandri E.L., Guzman C.A. (2023). Determinación Del Contenido de Aminoácidos En Harina de Quinoa de Origen Argentino. Evaluación Calid. Proteica.

[56] Craine E.B., Murphy K.M. (2020). Seed Composition and Amino Acid Profiles for Quinoa Grown in Washington State. Front. Nutr..

[57] Campos-Rodriguez J., Acosta-Coral K., Paucar-Menacho L.M., Campos-Rodriguez J., Acosta-Coral K., Paucar-Menacho L.M. (2022). Quinua (*Chenopodium quinoa*): Composición Nutricional y Componentes Bioactivos Del Grano y La Hoja, e Impacto Del Tratamiento Térmico y de La Germinación. Sci. Agropecu..

[58] Özer C.O., Seçen S.M. (2018). Effects of Quinoa Flour on Lipid and Protein Oxidation in Raw and Cooked Beef Burger during Long Term Frozen Storage. Food Sci. Technol..

[59] Torres O.L., Lema M., Galeano Y.V. (2021). Effect of Using Quinoa Flour (*Chenopodium quinoa* Willd.) on the Physicochemical Characteristics of an Extruded Pasta. Int. J. Food Sci..

[60] Ajbli N., Zine-eddine Y., Laaraj S., Ait El Alia O., Elfazazi K., Bouhrim M., Herqash R.N., Shahat A.A., Benbati M., Kzaiber F. (2025). Effect of Quinoa Flour on Fermentation, Physicochemical and Sensory Properties of Goat Milk Yogurt. Front. Sustain. Food Syst..

[61] Zhang J., Liu Y., Wang P., Zhao Y., Zhu Y., Xiao X. (2025). The Effect of Protein–Starch Interaction on the Structure and Properties of Starch, and Its Application in Flour Products. Foods.

[62] Jebalia I., Maigret J.E., Réguerre A.L., Novales B., Guessasma S., Lourdin D., Della Valle G., Kristiawan M. (2019). Morphology and Mechanical Behaviour of Pea-Based Starch-Protein Composites Obtained by Extrusion. Carbohydr. Polym..

[63] Jerkovic A., Kriegel A.M., Bradner J.R., Atwell B.J., Roberts T.H., Willows R.D. (2010). Strategic Distribution of Protective Proteins within Bran Layers of Wheat Protects the Nutrient-Rich Endosperm. Plant Physiol..

[64] Pelgrom P.J.M., Boom R.M., Schutyser M.A.I. (2015). Method Development to Increase Protein Enrichment During Dry Fractionation of Starch-Rich Legumes. Food Bioproc. Technol..

[65] Haros C.M., Reguera M., Sammán N., Paredes-López O. (2023). Latin-American Seeds: Agronomic, Processing and Health Aspects.

[66] Elbendari A.M., Ibrahim S.S. (2025). Optimizing Key Parameters for Grinding Energy Efficiency and Modeling of Particle Size Distribution in a Stirred Ball Mill. Sci. Rep..

[67] Jiang L., Liu P., Zhang Y., Yang X., Wang H., Gui X. (2019). Design Boundary Layer Structure for Improving the Particle Separation Performance of a Hydrocyclone. Powder Technol..

[68] Fernando S., Manthey F.A. (2022). Effect of Different Mills on the Physical and Flow Properties of Selected Black Bean Flour Particle Size Fractions. Cereal Chem..

[69] AmanNejad M., Barani K. (2021). Effects of Ball Size Distribution and Mill Speed and Their Interactions on Ball Milling Using DEM. Miner. Process. Extr. Metall. Rev..

[70] Lu X., Huang Q., Xiao J., Wang Y. (2022). Milled Miscellaneous Black Rice Particles Stabilized Pickering Emulsions with Enhanced Antioxidation Activity. Food Chem..

[71] Kruszelnicka W., Opielak M., Ambrose K., Pukalskas S., Tomporowski A., Walichnowska P. (2022). Energy-Dependent Particle Size Distribution Models for Multi-Disc Mill. Materials.

[72] Barretto R., Buenavista R.M., Pandiselvam R., Siliveru K. (2022). Influence of Milling Methods on the Flow Properties of Ivory Teff Flour. J. Texture Stud..

[73] Jeulin M., Cahuc O., Darnis P., Laheurte R. (2022). A 6-Components Mechanistic Model of Cutting Forces and Moments in Milling. Forces Mech..

[74] Mete E., Haszard J., Perry T., Oey I., Mann J., Morenga L. (2021). Te Effect of Wholegrain Flour Particle Size in Bread on Glycaemic and Insulinaemic Response among People with Risk Factors for Type 2 Diabetes: A Randomised Crossover Trial. Nutrients.

[75] Ahmed J., Alazemi A., Ponnumani P., B B.T., Soliman M., Emmanuval L., Thomas N.M. (2024). Transformation of Quinoa Seeds to Nanoscale Flour by Ball Milling: Influence of Ball Diameter and Milling Time on the Particle Sizing, Microstructure, and Rheology. J. Food Eng..

[76] Ballester-Sánchez J., Gil J.V., Fernández-Espinar M.T., Haros C.M. (2019). Quinoa Wet-Milling: Effect of Steeping Conditions on Starch Recovery and Quality. Food Hydrocoll..

[77] Ribera-Castelló A., Haros C.M. (2023). Obtaining Quinoa Germ via Wet Milling and Extracting Its Oil via Cold Pressing. Biol. Life Sci. Forum.

[78] Mufari J.R., Miranda-Villa P.P., Calandri E.L. (2018). Quinoa Germ and Starch Separation by Wet Milling, Performance and Characterization of the Fractions. LWT.

[79] Ray A., Muhammed Muneer B.M., Sakhare S.D. (2024). Analysis of Quinoa Roller Millstreams: Physical, Chemical, and Functional Properties. J. Cereal Sci..

[80] Chen Y., Han X., Chen D.-L., Ren Y.-P., Yang S.-Y., Huang Y.-X., Yang J., Zhang L. (2024). Dry Ball-Milled Quinoa Starch as a Pickering Emulsifier: Preparation, Microstructures, Hydrophobic Properties and Emulsifying Properties. Foods.

[81] Aweya J.J., Sharma D., Bajwa R.K., Earnest B., Krache H., Moghadasian M.H. (2025). Ancient Grains as Functional Foods: Integrating Traditional Knowledge with Contemporary Nutritional Science. Foods.

[82] Kumar Pathak M., Patel T. (2023). Quinoa: A History of Ancient Grains and Modern Diets. Agric. Food E-Newsl..

[83] Ma Y., Wu D., Guo L., Yao Y., Yao X., Wang Z., Wu K., Cao X., Gao X. (2022). Effects of Quinoa Flour on Wheat Dough Quality, Baking Quality, and in Vitro Starch Digestibility of the Crispy Biscuits. Front. Nutr..

[84] Wang Q., Li L., Zheng X. (2020). A Review of Milling Damaged Starch: Generation, Measurement, Functionality and Its Effect on Starch-Based Food Systems. Food Chem..

[85] Sánchez Y.G., Loubes M.A., González L.C., Tolaba M.P. (2024). Energy-Size Relationship and Starch Modification in Planetary Ball Milling of Quinoa. J. Cereal Sci..

[86] Tang B., Cheng B., Song X., Ji H., Li Y., Wang Z. (2024). Experimental Study on the Influence of Rotational Speed on Grinding Efficiency for the Vertical Stirred Mill. Minerals.

[87] Guo W., Gao P., Tang Z., Han Y., Meng X. (2021). Effect of Grinding Media Properties and Stirrer Tip Speed on the Grinding Efficiency of a Stirred Mill. Powder Technol..

[88] Madhumathi R., Prashanth K.V.H., Inamdar A.A. (2025). Fractionation of Roller-Milled Quinoa: Evaluation of Functional and Nutritional Properties of Different Fractions. J. Food Meas. Charact..

[89] Zhao F., Jing L., Wang D., Bao F., Lu W., Wang G. (2018). Grain and Starch Granule Morphology in Superior and Inferior Kernels of Maize in Response to Nitrogen. Sci. Rep..

[90] Salvador-Reyes R., Rebellato A.P., Lima Pallone J.A., Ferrari R.A., Clerici M.T.P.S. (2021). Kernel Characterization and Starch Morphology in Five Varieties of Peruvian Andean Maize. Food Res. Int..

[91] Xu Y. (2018). The Fractal Evolution of Particle Fragmentation under Different Fracture Energy. Powder Technol..

[92] Marc R.A., Díaz A.V., Izquierdo G.D.P. (2020). Food Processing.

[93] Tian X., Wang X., Ma S., Sun B., Li L., Wang Z. (2022). Study of the Ball Milling Condition Effect on Physicochemical and Structural Characteristics of Wheat Flour. J. Food Process Preserv..

[94] Huang Y., Pu D., Hao Z., Yang X., Zhang Y. (2021). The Effect of Prickly Ash (*Zanthoxylum bungeanum* Maxim) on the Taste Perception of Stewed Sheep Tail Fat by Lc-Qtof-Ms/Ms and a Chemometrics Analysis. Foods.

[95] Chai Y., Zhong W., Yang C., Klahn E., Grosshans H. (2019). Modeling the Agglomeration of Electrostatically Charged Particles. J. Phys. Conf. Ser..

[96] FAO/WHO Platform of Information on Quinoa. https://www.fao.org/in-action/quinoa-platform/en/.

[97] Contreras-Jiménez B., Torres-Vargas O.L., Rodríguez-García M.E. (2019). Physicochemical Characterization of Quinoa (*Chenopodium quinoa*) Flour and Isolated Starch. Food Chem..

[98] Bangar S.P., Singh A., Ashogbon A.O., Bobade H. (2023). Ball-Milling: A Sustainable and Green Approach for Starch Modification. Int. J. Biol. Macromol..

[99] Junejo S.A., Wang J., Liu Y., Jia R., Zhou Y., Li S. (2022). Multi-Scale Structures and Functional Properties of Quinoa Starch Extracted by Alkali, Wet-Milling, and Enzymatic Methods. Foods.

[100] Sharma S., Kataria A., Singh B. (2022). Effect of Thermal Processing on the Bioactive Compounds, Antioxidative, Antinutritional and Functional Characteristics of Quinoa (*Chenopodium quinoa*). LWT.

[101] Nogueira-de-Almeida C.A., Ribas Filho D. (2021). Positioning on the Use of Polyols as Table Sweeteners. Int. J. Nutrol..

[102] Sharma A., Kashyap S., Singh S. (2025). Exploring the Advances in Quinoa Processing: A Comprehensive Review Enhancing Nutritional Quality and Health Benefits along with Industrial Feasibility of Quinoa. Food Res. Int..

[103] Pereira E., Encina-Zelada C., Barros L., Gonzales-Barron U., Cadavez V., Ferreira I.C. (2019). Chemical and Nutritional Characterization of *Chenopodium quinoa* Willd (Quinoa) Grains: A Good Alternative to Nutritious Food. Food Chem..

[104] Romano N., Ureta M.M., Guerrero-Sánchez M., Gómez-Zavaglia A. (2020). Nutritional and Technological Properties of a Quinoa (*Chenopodium quinoa* Willd.) Spray-Dried Powdered Extract. Food Res. Int..

[105] Coţovanu I., Mironeasa C., Mironeasa S. (2023). Nutritionally Improved Wheat Bread Supplemented with Quinoa Flour of Large, Medium and Small Particle Sizes at Typical Doses. Plants.

[106] Lamothe L.M., Srichuwong S., Reuhs B.L., Hamaker B.R. (2015). Quinoa (*Chenopodium quinoa* W.) and Amaranth (*Amaranthus caudatus* L.) Provide Dietary Fibres High in Pectic Substances and Xyloglucans. Food Chem..

[107] Jiang F., Du C., Guo Y., Fu J., Jiang W., Du S. (2020). kui Physicochemical and Structural Properties of Starches Isolated from Quinoa Varieties. Food Hydrocoll..

[108] Li G., Zhu F. (2018). Quinoa Starch: Structure, Properties, and Applications. Carbohydr. Polym..

[109] Londoño-Restrepo S.M., Rincón-Londoño N., Contreras-Padilla M., Millan-Malo B.M., Rodriguez-Garcia M.E. (2018). Morphological, Structural, Thermal, Compositional, Vibrational, and Pasting Characterization of White, Yellow, and Purple Arracacha Lego-like Starches and Flours (*Arracacia xanthorrhiza*). Int. J. Biol. Macromol..

[110] Salman H., Blazek J., Lopez-Rubio A., Gilbert E.P., Hanley T., Copeland L. (2009). Structure–Function Relationships in A and B Granules from Wheat Starches of Similar Amylose Content. Carbohydr. Polym..

[111] Ma J., Hu J., Sha X., Meng D., Yang R. (2022). Phycobiliproteins, the Pigment-Protein Complex Form of Natural Food Colorants and Bioactive Ingredients. Crit. Rev. Food Sci. Nutr..

[112] González L.C., Loubes M.A., Tolaba M.P. (2018). Incidence of Milling Energy on Dry-Milling Attributes of Rice Starch Modified by Planetary Ball Milling. Food Hydrocoll..

[113] Bala S., Garg D., Thirumalesh B.V., Sharma M., Sridhar K., Inbaraj B.S., Tripathi M. (2022). Recent Strategies for Bioremediation of Emerging Pollutants: A Review for a Green and Sustainable Environment. Toxics.

[114] Cao H., Sun R., Liu Y., Wang X., Guan X., Huang K., Zhang Y. (2022). Appropriate Microwave Improved the Texture Properties of Quinoa Due to Starch Gelatinization from the Destructed Cyptomere Structure. Food Chem X.

